# Supplementary material for: Splice-Junction-Based Mapping of Alternative Isoforms in the Human Proteome
Source: Cell Rep. Author manuscript; Available in PMC 2020 Jan 15. (PMC6961840; doi:10.1016/j.celrep.2019.11.026)
Supplement: 2 [file NIHMS1546469-supplement-2.zip › DF1/PXD010154/Heart/Heart_21_TTN_ASNEYGSVSCTATLTVTEPPKFVK.pdf]

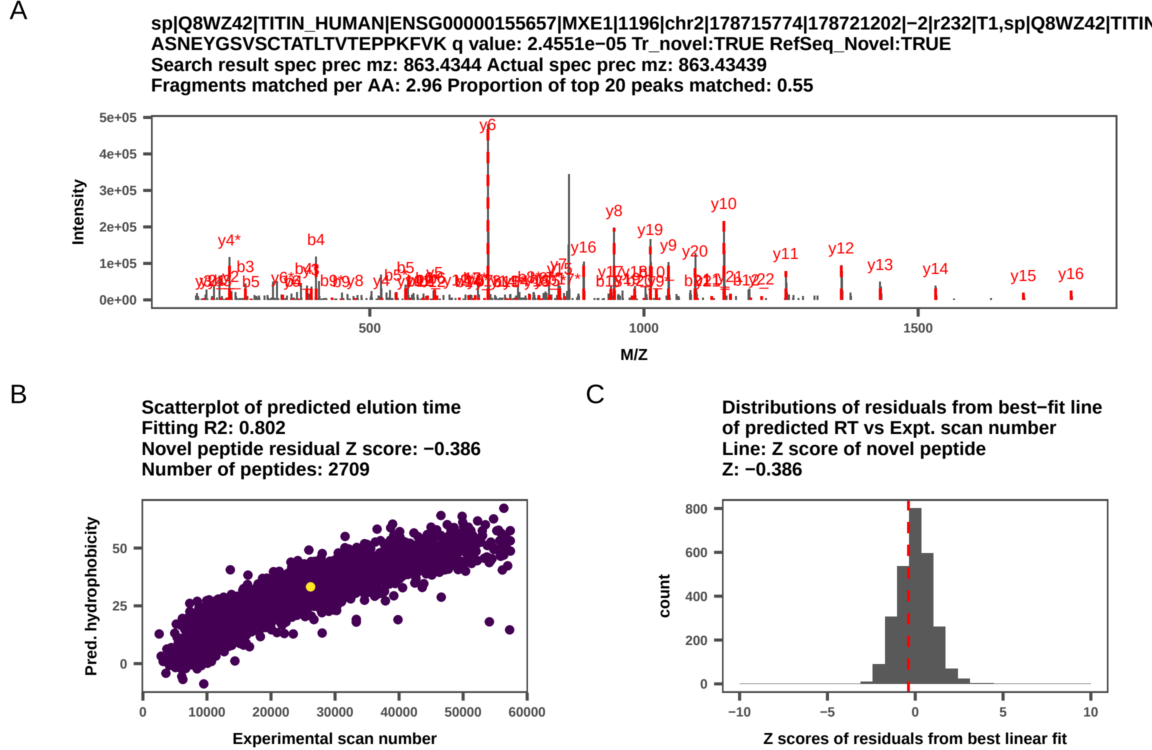
A
sp|Q8WZ42|TITIN_HUMAN|ENSG00000155657|MXE1|1196|chr2|178715774|178721202|−2|r232|T1,sp|Q8WZ42|TITIN
ASNEYGSVSCTATLTVTEPPKFVVK q value: 2.4551e−05 Tr_novel:TRUE RefSeq_Novel:TRUE
Search result spec prec mz: 863.4344 Actual spec prec mz: 863.43439
Fragments matched per AA: 2.96 Proportion of top 20 peaks matched: 0.55
Intensity
5e+05
4e+05
3e+05
2e+05
1e+05
0e+00
y6
y8
y10
y19
y9
y20
y16
y11
y12
y13
y14
y15
y16
y4*
b4
b3
b5
y8
500
1000
1500
M/Z
B
Scatterplot of predicted elution time
Fitting R2: 0.802
Novel peptide residual Z score: −0.386
Number of peptides: 2709
Pred. hydrophobicity
50
25
0
0
10000
20000
30000
40000
50000
60000
Experimental scan number
C
Distributions of residuals from best−fit line
of predicted RT vs Expt. scan number
Line: Z score of novel peptide
Z: −0.386
count
800
600
400
200
0
−10
−5
0
5
10
Z scores of residuals from best linear fit
